# Biochar Administration to San Marzano Tomato Plants Cultivated Under Low-Input Farming Increases Growth, Fruit Yield, and Affects Gene Expression

**DOI:** 10.3389/fpls.2020.01281

**Published:** 2020-08-27

**Authors:** Maria Tartaglia, Simona Arena, Andrea Scaloni, Mauro Marra, Mariapina Rocco

**Affiliations:** ^1^Department of Science and Technology, University of Sannio, Benevento, Italy; ^2^Proteomics & Mass Spectrometry Laboratory, ISPAAM, National Research Council, Naples, Italy; ^3^Department of Biology, University of Tor Vergata, Rome, Italy

**Keywords:** biochar, tomato, proteomics, climate change, low-input farming

## Abstract

Biochar is a rich-carbon charcoal obtained by pyrolysis of biomasses, which was used since antiquity as soil amendant. Its storage in soils was demonstrated contributing to abate the effects of climate changes by sequestering carbon, also providing bioenergy, and improving soil characteristics and crop yields. Despite interest in this amendant, there is still poor information on its effects on soil fertility and plant growth. Considerable variation in the plant response has been reported, depending on biomass source, pyrolysis conditions, crop species, and cultivation practices. Due to these conflicting evidences, this work was aimed at studying the effects of biochar from pyrolyzed wood at 550°C, containing 81.1% carbon and 0.91% nitrogen, on growth and yield of tomato plants experiencing low-input farming conditions. San Marzano ecotype from Southern Italy was investigated, due to its renowned quality and adaptability to sustainable farming practices. Biochar administration improved vegetative growth and berry yield, while affecting gene expression and protein repertoire in berries. Different enzymes of carbon metabolism and photosynthesis were over-represented, whereas various stress-responsive and defense proteins were down-represented. Molecular results are here discussed in relation to estimated agronomic parameters to provide a rationale justifying the growth-promoting effect of this soil amendant.

## Introduction

While World’s food demand is raising as a result of the rapidly growing of population, increasing atmospheric CO_2_ concentration and global climate changes, due to anthropogenic activities, greatly impact on agriculture, reducing crop yields, and decreasing the availability and the quality of soils in terms of water and nutrient content or heavy metal contamination (Lafferty, 2009; Auffhammer et al., 2012). In agricultural soils, the addition of amendants rich in organic carbon has been proposed as a sustainable remediation to improve soil fertility and increase crop productivity (Marris, 2006; Lehmann, 2007a). This practice also provides a mean to permanently sequester carbon, thereby reducing CO_2_ release in the atmosphere and mitigating global climate changes. Biochar is a carbon-rich product produced by pyrolysis of biomasses of different origin, under high-temperature and low-oxygen conditions (Lehmann, 2007b; Laird et al., 2009). Due to its peculiar structural features, like porosity, high surface area of particles and affinity for charged compounds (Keech et al., 2005), biochar has been proposed for different purposes, such as soil management, feedstuff for livestock, and water purification. Recently, the renewed attention to sustainable practices in agriculture prompted extensive use of biochar to increase the fertility of soils and to improve the productivity of crop plants (Biederman and Stanley Harpole, 2013; Laghari et al., 2016). In fact, the addition of biochar to the soil improves its water and nutrient retention capacity (Laird et al., 2010), increases bioavailability of phosphate and potassium (Laghari et al., 2016), and stimulates soil microbial activity (Steinbeiss et al., 2009); conversely, it decreases that of heavy metals (Park et al., 2011) and N_2_O emission. Although negligible to adverse effects have also been reported (Spokas and Reicosky, 2009; Jeffery et al., 2011; Spokas et al., 2011), recently, comprehensive meta-analyses of published studies demonstrated that biochar, on the overall, has a positive effect on ecosystems and cultures in terms of plant productivity, nutrient uptake, and soil properties (Jeffery et al., 2011; Biederman and Stanley Harpole, 2013). Contradictory results have also been obtained in studies aimed at testing the effectiveness of biochar as a primer of plant defense responses to pathogens. In fact, a reduction of the severity of infection has been reported for some fungine foliar diseases, such as powdery mildew, anthracnose, or gray mold (Elad et al., 2011; Harel et al., 2012; Mehari et al., 2015), and for nematode root infections (Huang et al., 2015), whereas inconsistent or negative effects have been observed in other root-pathogen interactions (Elmer and Pignatello, 2011; Akhter et al., 2015; Shoaf et al., 2016) or foliar diseases (Copley et al., 2017).

The effects of biochar seem greatly influenced by the feedstock used to produce it and by conditions of pyrolysis (Méndez et al., 2013), which can impact on structure, nutrient and phenolic content, and pH value of the final product (Novak et al., 2009). Moreover, the effects of biochar on plant cultivations also vary in dependence of the nature (type, mineral, and nutrient content) and conditions (fertilization and humidity) of the soil to which it is added (Van Zwieten et al., 2010a; Van Zwieten et al., 2010b; Biederman and Stanley Harpole, 2013). Tomato (*Lycopersicon esculentum*, Mill.) is one of the most economically important vegetable crop, especially in the Mediterranean area, and has been used as a model crop species for genomics and proteomics studies (Rocco et al., 2006; Sant’Ana and Lefsrud, 2018). Nowadays, information on the effect of biochar administration to tomato cultivation in terms of growth, yield and quality is very poor. In a field experiment, Vaccari and coworkers reported that biochar addition to a fertile soil improved tomato (Pietrarossa cultivar) growth but not yield (Vaccari et al., 2015). Polzella et al. reported that biochar administration to tomato plants (San Marzano ecotype) in a neutral and low in nutrients soil did not significantly improved growth and yield performances; results by proteomic and qRT-PCR analysis pointed to a limited impact of biochar on photosynthesis and defense genes (Polzella et al., 2019).

Considering the above-reported variability in plant productivity and the scarce information concerning tomato growth and yield performances as a result of biochar administration, this work was aimed at investigating the impact of biochar produced from wood under controlled conditions (550°C pyrolysis temperature, 81.1% carbon, 0.91% nitrogen) on the aboveground growth and productivity of tomato plants of the S. Marzano ecotype cultivated in an acidic soil, under low-input conditions. The San Marzano ecotype was chosen for this study because it is a traditional cultivar from Campania, South Italy, which has become a top quality variety owing to its peculiar organoleptic traits (Ercolano et al., 2008; Ercolano et al., 2014); furthermore, as local accession, it is also well suited to low-input cultivation or organic farming (Negri, 2003). Agronomic parameters such as height and number of flower buds, and number and weight of berries were evaluated and related to molecular data obtained from qRT-PCR and proteomic analyses.

## Materials and Methods

### Plant Material

Verfofood biochar was purchased from Green Biochar, Torino, Italy. This fine grain coal is produced from wood at a maximum pyrolysis temperature of 550°C. It contains 81.1% of carbon and 0.91% nitrogen; it also has a pH value of 8.21 and an ash content of 7.74%. The soil matrix used for the growth of tomato plants was a typical Mediterranean agriculturally managed soil, classified as Eutric Cambisol collected in Campania, Italy. After sampling, the soil was dried at 40°C for 24 h, and sieved (<2 mm). The soil pH in water was 5.6 and its Water Holding Capacity (WHC) and ash content was 35.85% and 88%, respectively. Seeds of the 204-San Marzano 2 accession (Consorzio Agrario, Parma, Italy) were sowed in plastic pots (10 l) containing soil or soil with 5% of biochar. Ten seeds each pot were sowed and five pots containing soil plus five pots containing soil and 5% biochar were prepared; all pots were irrigated with the same volume of water two times a week. Fifteen days after sowing, a single plant for each pot was selected to continue the experiment, and pots were placed in greenhouse, under controlled conditions, with 14 h of light/day. After 50 days of growth, agricultural parameters, like height and number of flowers buds, were estimated every 15 days. At the end of the experiment (177 days after sowing), tomato fruits from each pot were collected and their number and weight registered. Immediately after collection, fruits were cut longitudinally into four parts and frozen in liquid N_2_ following seeds removal. Fruits were stored at −80°C until their use.

### Protein Extraction and 2-D Electrophoresis

Fruit samples (2.5 g of frozen tomato fruits) were powdered in a mortar using liquid N_2_, and suspended in 7.5 ml of extraction buffer (700 mM sucrose, 500 mM Tris-HCl, pH 7.5, 50 mM EDTA, 100 mM KCl, 2% w/v β-mercaptoethanol, and 1 mM PMSF) for 15 min, on ice. After the addition of an equal volume of Tris-saturated phenol (500 mM Tris-HCl, pH 7.5), the mixture was vortexed for 10 min and then centrifuged at 10,000 × *g*, for 15 min, at 4°C. The upper phenol phase was removed and extracted twice with the extraction buffer. Proteins were precipitated from the phenol phase by the addition of five volumes of saturated ammonium acetate in methanol, overnight at −20°C. Precipitated proteins were centrifuged at 10,000 × *g*, for 30 min (Rocco et al., 2006).

Protein pellets were washed once with ice-cold methanol and three times with ice-cold acetone, dried and solved in IEF buffer (9 M urea, 4% w/v CHAPS, 0.5% v/v Triton X-100, 20 mM DTT, and 1% w/v carrier ampholytes pH 3–10) (BioRad, Hercules, CA, USA). Protein concentration was quantified using the BioRad protein assay, using BSA as a standard. IPG strips (17 cm, pH 4–7, BioRad ReadyStrip, BioRad) were rehydrated with 300 µl of IEF buffer containing 400 µg of total proteins, overnight. Proteins were focused using a Protean IEF Cell (BioRad, Segrate MI, Italy) at 12°C, applying 250 V (90 min), 500 V (90 min), 1,000 V (180 min), and 8,000 V, for a total of 53 KVh. After focusing, proteins were reduced by incubating the IPG strips with 1% w/v DTT in 10 ml of 50 mM Tris-HCl (pH 8.8), 6 M urea, 30% w/v glycerol, 2% w/v SDS, and a dash of bromophenol blue, for 15 min, and alkylated with 2.5% w/v iodoacetamide in 10 ml of the same buffer, for 15 min. Electrophoresis in the second dimension was carried out using a Protean apparatus (BioRad, Segrate MI, Italy) and 12% polyacrylamide gels in 25 mM Tris (pH 8.3), 1.92 M glycine and 1% w/v SDS, with 120 V applied for 12 h. Gels were stained with colloidal Coomassie G-250. Analyzes were done on two technical replicates for three biological samples.

### Image Acquisition and Analysis

2-DE gel images were acquired using a GS-800 calibrated densitometer (BioRad, Segrate MI, Italy). Image analysis was performed using the PD Quest software (BioRad, Segrate MI, Italy). Spot detection and matching between gels were performed automatically, followed by manual verification. Protein spots were annotated only if detectable in all gels. After normalization of the spot densities against the whole gel densities, the percentage volume of each spot was averaged for six different (two replicates of three samples) gels; Student’s t-test analysis (p < 0.05) was performed to find out statistically significant spot volume fold changes (>1.5 or <0.66) associated with biochar presence in soil.

### Spot Digestion, Mass Spectrometric Analysis, and Protein Identification

Spots from 2-DE were excised from the gel and shattered. Proteins were *in-gel* reduced with dithiothreitol, S-alkylated with iodoacetamide, and then digested with trypsin. Resulting peptide mixtures were subjected to a desalting/concentration step on μZip-TipC_18_ devices (Millipore, Bedford, MA, USA) before MS analysis. Recovered peptides were then analyzed for protein identification by nanoLC-ESI-LIT-MS/MS, using an LTQ XL mass spectrometer (Thermo Fisher Scientific, USA) equipped with a Proxeon nanospray source connected to an Easy-nanoLC (Proxeon, Denmark). Peptides were resolved on an Easy C_18_ column (100 mm × 0.075 mm, 3 μm) (Proxeon) (Paiardini et al., 2014). Mobile phases were 0.1% v/v formic acid (FA) (solvent A) and 0.1% v/v FA in ACN (solvent B), running at a total flow rate of 300 nl/min. Linear gradient was initiated 20 min after sample loading; solvent B ramped from 5 to 35% over 45 min, from 35% to 60% over 10 min, and from 60% to 95% over 20 min. Spectra were acquired in the range m/z 400–2000. Each peptide mixture was analyzed under CID-MS/MS data-dependent product ion scanning procedure, enabling dynamic exclusion (repeat count 1 and exclusion duration 60 s), over the three most abundant ions. Mass isolation window and collision energy were set to *m/z* 3 and 35%, respectively.

Raw nanoLC-ESI-LIT-MS/MS data were searched with v.2.2.06 MASCOT software (Matrix Science, UK) against an updated (07/2017), non-redundant UniProtKB database (taxonomy *Viridiplantae*) to identify protein(s) present within each gel spot. Database searching was performed by using Cys carbamidomethylation and Met oxidation as fixed and variable protein modifications, respectively, a mass tolerance value of 1.8 Da for precursor ion and 0.8 Da for MS/MS fragments, trypsin as proteolytic enzyme, and a missed cleavage maximum value of 2. Other MASCOT parameters were kept as default. Protein candidates assigned on the basis of at least two sequenced peptides with an individual peptide expectation value <0.05 (corresponding to a confidence level for peptide identification >95%) were considered confidently identified. Definitive peptide assignment was always associated with manual spectra visualization and verification.

### RNA Extraction and cDNAs Synthesis

The MirPremier microRNA isolation kit (Sigma-Aldrich, Milan, Italy) was used to extract RNA from tomato berry samples. The samples (0.1-g fresh weight) were homogenized in liquid N_2_, with mortar and pestle, and aliquoted (0.07 g) into a pre-cooled tube containing 750 μl of kit lysis solution adding 1% of v/v β-mercaptoethanol. After 5-min incubation at 55°C, samples were centrifuged at 14,000 × *g*, and the supernatant loaded onto a filtration column included in the kit. The filtrate was recovered by centrifugation at 14,000 × *g*, for 1 min, which was added with 1.5 vol of binding solution from the same kit. The mixture was loaded onto the binding column, and the corresponding filtrate removed by centrifugation at 14,000 × *g*, for 1 min. To remove any traces of phospholipids, proteins, and carbohydrates, the column was washed with 700 μl of pure ethanol, 500 μl of binding solution and 500 μl of pure ethanol. In order to elute nucleic acids, 30 μl of nuclease-free H_2_O were added to the column and the eluate was recovered by centrifugation at 14,000 × *g*, for 1 min. To degrade genomic DNA and obtain pure RNA, we used the RNeasy/QIamp columns and RNase-free DNase set (Quiagen, Milan, Italy), following the manufacturer’s instructions. Extracted RNA was retrotranscribed to cDNA by using the ImProm-II Reverse Transcription System kit (Promega, Milan, Italy) and the mini thermal cycler BioRad (Segrate MI, Italy). To 10 μl of purified RNA, 1 μl of Primer Oligo (dt) s and 1 μl of dNTP were added; the mixture was incubated in the thermal cycler at 70°C, for 5 min. After the addition of 8 μl of master mix (20 μl Improm-II 5X reaction buffer, 10 μl MgCl_2_, 5 μl Recombination RNasin ribonuclease inhibitor and 5 μl Improm-II Reverse Trascriptase), the mixture was incubated again in the thermal cycler at 25°C, for 5 min, at 50°C for 60 min, and at 70°C, for 15 min. After retrotranscription, the samples were stored at −20°C.

### RT-qPCR

Gene primers were selected according to (Viger et al., 2015) and designed using the NCBI Primer Blast tool; their nature is reported in **Table 1**. For RT-qPCR, the EvaGreen 2X qPCR MasterMix-R kit (Applied Biological Materials Vancouver, Canada) was used. A 7300 Real-time PCR system (Applied Biosystems) was set to perform an initial denaturation at 95°C for 1 min, an annealing phase at 95°C, for 5 min, and 40 subsequent cycles of denaturation at 95°C, for 30 s, annealing at 60°C, for 30 s, and extension at 72°C, for 30 s. Relative gene expression quantification was carried out using the 2-ΔΔCt method (Livak and Schmittgen, 2001). Experiments were carried out in triplicate for each biological sample.

**Table 1:** Forward and reverse primer sequences designed and used for RT-qPCR experiments.

Primer	Sequence
Nb BETA ACTIN F	TGGACTCTGGTGATGGTGTC
Nb BETA ACTIN R	CCTCCAATCCAAACACTGTA
PDF1.2a F	GCTGCTTTCGGTGAGTAATAATG
PDF1.2a R	CCATGTCCCACTTGGCTTCT
PDF1.2b F	GCAGCTTTTGGTTAGTAATGCTCT
PDF1.2b R	AGTACCACTTGGCTTCTCGC
JAZ F	AGCCAACAAACAGAACCCCA
JAZ R	AATTCCGTCTCGCGATTGGT
LOX F	GCCTCAATTGTCGATGGTGC
LOX R	TCGTTGCGATCCCAGTCAAA

### Data Analysis

Agronomic parameter data are reported as mean ± SD of three independent experiments. Statistical analysis was performed by ANOVA followed by the Student-Newman-Keulus test, with the minimum level of significance being p < 0.05. Statistical significance of differences in protein spot densities from densitometric analysis of 2D-electrophoretic gels was assessed automatically by PD Quest software (Bio-Rad), using the Student’s t test (p < 0.05).

## Results and Discussion

### Effect of Biochar Addition on Soil Characteristics

Analysis of soil with and without the addition of biochar for corresponding pH, and carbon (C), phosphorus (P), potassium (K), magnesium (Mg), and total nitrogen (N) content values is reported in **Table 2**. These results demonstrated that biochar addition highly modified the pH value as well as C and N content of soil, while it poorly affected the other constitutive soil parameters.

**Table 2:** Chemical parameters of soil samples with and without biochar addition.

**Sample**	**pH**	**C (%)**	**N (%)**	**P (%)**	**K (%)**	**Mg (%)**
Soil	5.62 ± 0.02	3.20 ± 0.01	0.16 ± 0.01	0.26 ± 0.01	1.62 ± 0.01	0.75 ± 0.01
Biochar	8.21 ± 0.03	81.10 ± 0.02	0.91 ± 0.02	0.08 ± 0.01	0.88 ± 0.01	0.41 ± 0.01
Soil + biochar	7.10 ± 0.02	17.81 ± 0.02	1.21 ± 0.02	0.27 ± 0.01	1.74 ± 0.01	0.89 ± 0.01

Data on corresponding pH value, and carbon (C), phosphorus (P), potassium (K), magnesium (Mg), and total nitrogen (N) percentage content are shown for comparison.

### Effect of Biochar on Growth and Productivity of Tomato Plants

**Figure 1:**
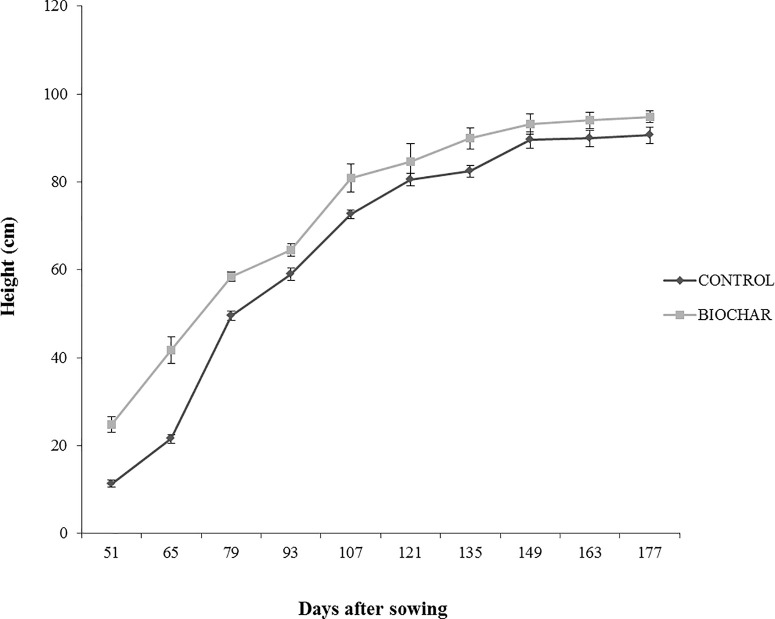
Average height (in cm) of tomato plants experiencing low-input farming conditions in soils amended with or not with 5% Verfofood biochar. Measurements were performed the 50th day after sowing using one sample per five pots.

**Figure 2:**
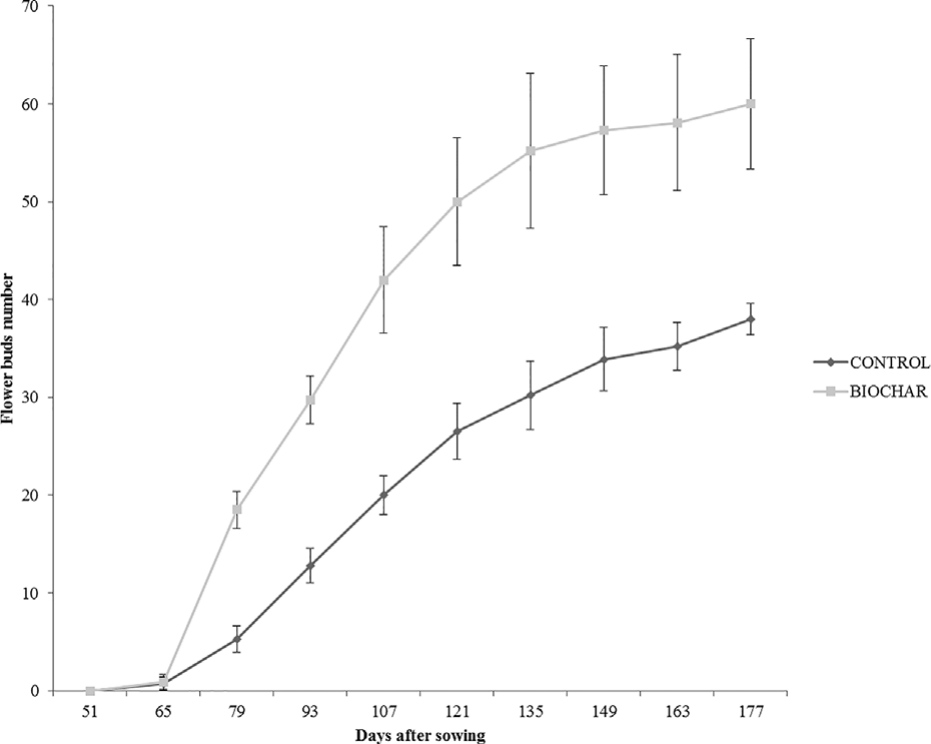
Average number of flower buds in tomato plants experiencing low-input farming conditions in soils amended with or not with 5% Verfofood biochar. Measurements were performed the 50th day after sowing using one sample per five pots.

**Figure 3:**
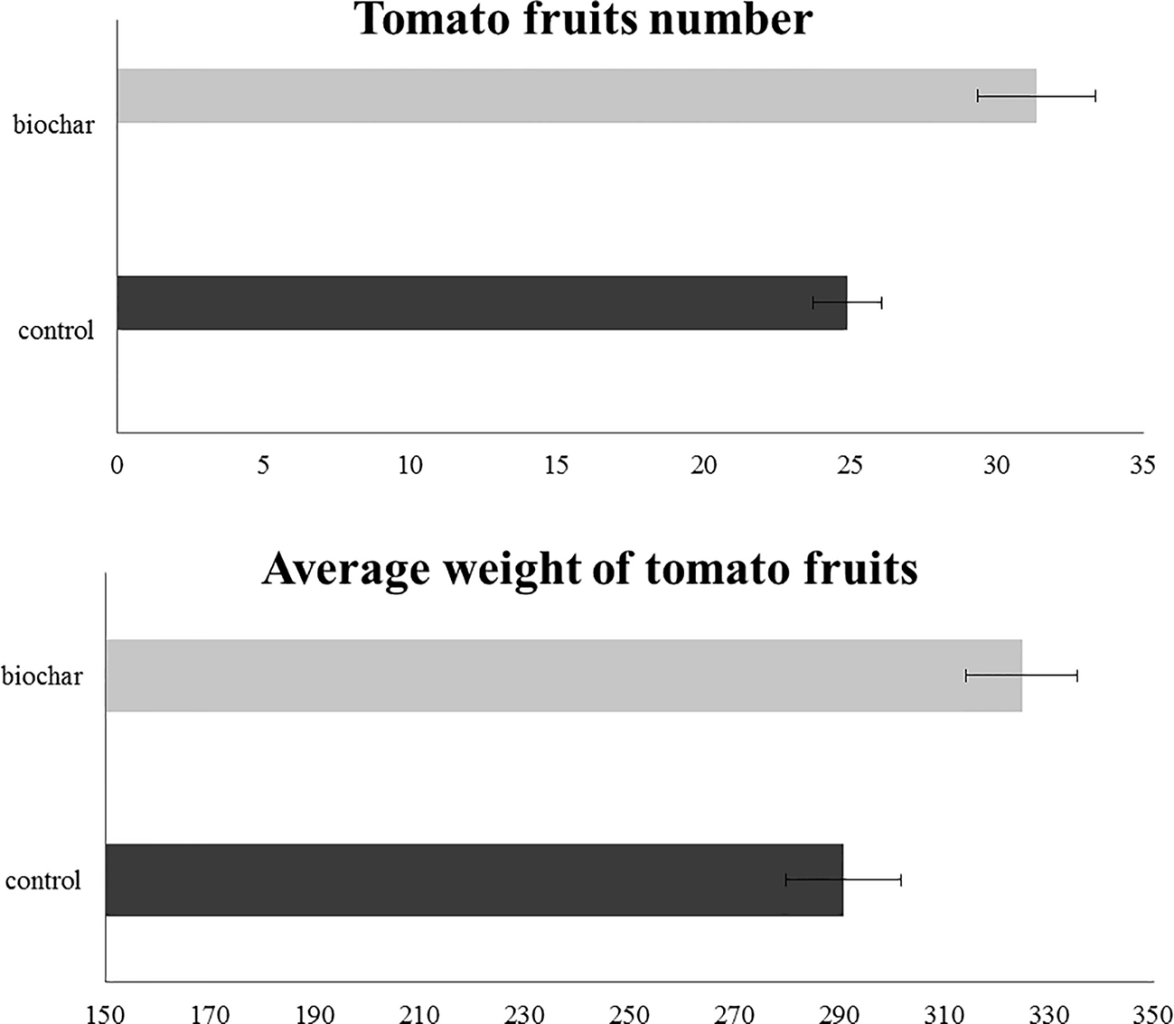
Fruit characteristics in tomato plants experiencing low-input farming conditions in soils amended with or not with 5% Verfofood biochar. **(A)** Average number of fruits per plant at the end of the experiment. **(B)** Average weight of the fully-ripen fruits. Measurements were performed at the end of plant treatment using one sample per five pots.

In order to evaluate the effect of a 5% biochar administration on the aboveground growth and productivity of San Marzano tomato plants, the corresponding height and the number of flower buds were measured every 14 days, starting from the 50th day after sowing (**Figure 1**). Similarly, the average number and weight of ripe berries was estimated at the end of the growth period (177 days after sowing) (**Figure 2**). Results demonstrated that biochar administration accelerated the growth of treated plants, particularly in the early phases of growth, whereas the effect was reduced at the end of the growing period (**Figure 1**). On the other hand, treated plants showed a marked increase in the number of floral buds at every stage of the cultivation, a parameter that is strictly related to their productivity (**Figure 2**). In fact, the average number (**Figure 3A**) and weight (**Figure 3B**) of ripe berries at the end of cultivation period resulted markedly increased in San Marzano plants treated with 5% biochar, when compared to untreated plants. These results demonstrated that biochar has a marked effect on tomato plant growth and productivity, when this specific biochar type and plants experiencing low-input farming conditions are considered.

### Proteomic Analysis of Tomato Berries From Plants Grown in the Presence of Biochar

**Figure 4:**
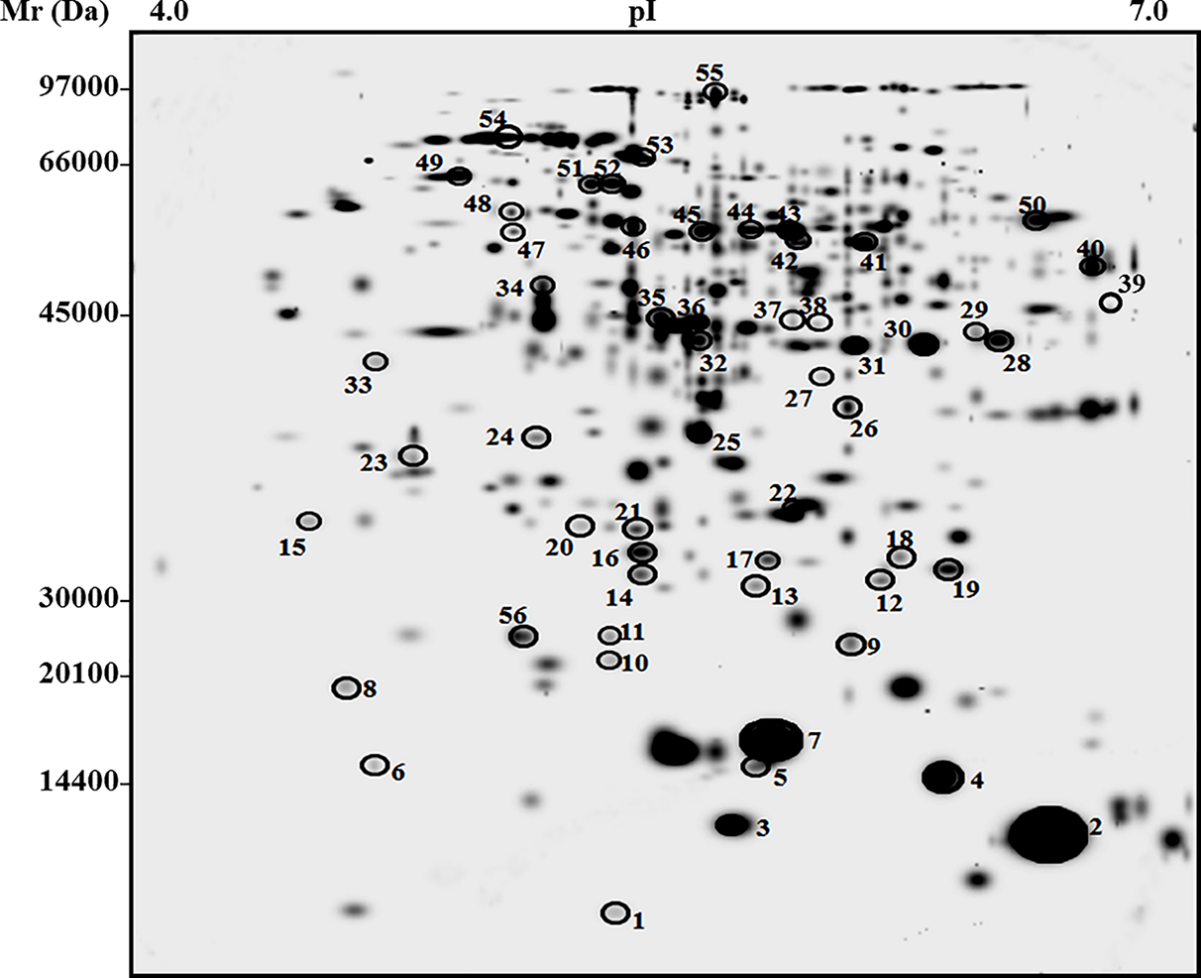
Two-dimensional electrophoretic reference map of fruits from tomato plants experiencing low-input farming conditions in soils amended with or not with 5% Verfofood biochar. Proteins were resolved on IPGs (first dimension) and 12% SDS-PAGE (second dimension) and were visualized by colloidal Coomassie blue staining. Spot numbering coincides with that reported in **Table 3**; experiments were carried out in triplicate for each biological sample.

Differential proteomic characterization of tomato fruits at final maturation stage from San Marzano plants grown in soil amended with 5% biochar and control soil was achieved by 2-DE analysis of corresponding proteins resolved in the pI range 4–7 (first dimension) and the mass range 10–150 kDa (second dimension), followed by gel staining with colloidal Coomassie blue (see experimental section for details). This analysis allowed a comparison of the protein repertoire of biochar-amended and control samples to determine statistically significant quantitative variations due to biochar addition to soil. In particular, average proteomic maps for berries from biochar-treated and control plants showed 875 and 805 reproducible spots, respectively, with a similarity degree of 68%. The master gel of this proteomic analysis is shown in **Figure 4**. To ascertain quantitative changes in corresponding proteomic maps, relative spot densities were evaluated by software-assisted analysis. Statistical analysis (p > 0.05) revealed 56 protein spots as differentially represented between biochar-amended and control samples. Differentially represented spots were excised from gels, trypsinolyzed, and subjected to nanoLC-ESI-LIT-MS/MS analysis for protein identification. The list of the identified proteins, together with their quantitative variations between Biochar amended and control plants, is reported in **Table 3**. Functional categorization according to Gene Ontology annotation and literature data allowed grouping identified proteins into two main functional categories, namely, energy/carbon metabolism and stress/defense. Different proteins with uncharacterized function or not grouping in the above categories were also identified. Above-mentioned groups are discussed in the dedicated sections reported below.

**Table 3:** Differentially represented proteins in tomato fruits from plants treated with 5% Verfofood biochar as identified by combined 2-DE and nanoLC-ESI-LIT-MS/MS of corresponding spot digests.

**Spot**	**Protein description**	**UniProtKBaccession**	**Gene name**	**Organism**	**Unique peptides**	**Sequence coverage (%)**	**pI_th_/Mw_th_**	**Fold change(biochar vs. control)**		
***Energy and carbon metabolism***									
1	Ribulose bisphosphate carboxylase small chain 2A	P07179	RBCS-2A	SL	7	34	6.58/20278		4.6
8	Chloroplast inorganic pyrophosphatase	K4B2L1	PPA6	SL	3	11	6.01/32383		1.9
12	Probable adenylate kinase 2	Q9FIJ7	KAD2	AT	5	19	7.11/31452		3.5
16	Triose phosphate isomerase	K4B3X5	TIM	SL	31	48	6.45/34665		2.2
18	Triose phosphate isomerase cytosolic isoform	Q6T379	TPIP1	SC	12	60	5.73/27040		2.2
20	Triose phosphate isomerase	K4B3X5	TIM	SL	5	21	6.45/34665		2.3
21	Soluble inorganic pyrophosphatase	K4B2L1	PPA6	SL	3	11	6.01/32383		1.6
24	Oxygen-evolving enhancer protein 1-2	K4BCF4	PSBO2	SL	3	17	5.84/35271		2.3
28	Malate dehydrogenase cytosolic	K4CW40	MDH1	SL	6	15	5.91/35384		3.3
29	Glyceraldehyde 3-phosphate dehydrogenase	K4BYG6	GAPC2	SL	7	14	6.34/36651		7.8
30	Mitochondrial NAD-dependent malate dehydrogenase	Q8L5A6	mMDH	ST	15	33	8.87/35661		3.5
31	Malate dehydrogenase cytosolic	K4CW40	MDH1	SL	19	23	5.91/35384		3.9
32	Ribulose bisphosphate carboxylase/oxygenase activase	O49074	RCA	SPE	5	12	8.61/50701		2.4
35	Ribulose bisphosphate carboxylase/ oxygenase activase	O49074	RCA	SPE	7	18	8.61/50701		5.1
36	Ribulose biphosphate carboxylase/oxygenase activase	O49074	RCA	SPE	8	16	8.61/50701		5.4
37	Enolase	P26300	PGH1	SL	10	25	5.68/47798		2.3
38	Phosphoglycerate kinase	K4CHY3	T8K14.3	SL	13	40	5.78/42489		3.1
39	Fructose-bisphospate aldolase	Q9FUG7	SCA1	FA	2	23	6.93/38515		2.0
40	Ribulose bisphosphate carboxylase large chain	P48698	rbcL	DS	18	36	6.54/51948		3.0
41	UTP-glucose-1-phosphate uridylyltransferase	K4D601	UGP2	SL	41	48	5.84/51819		2.2
42	Enolase	P26300	PGH1	SL	53	59	5.68/47798		2.2
43	Enolase	P26300	PGH1	SL	46	49	5.68/47798		2.0
44	ATP synthase β-subunit	K4BX20	ATPB	SL	38	21	5.74/59607		2.5
45	ATP synthase β-subunit	K4BX20	ATPB	SL	64	19	5.74/59607		2.3
46	ATP synthase CF1 beta subunit	A0A0C5CEC7	atpB	SL	21	44	5.28/53467		5.8
47	ATP synthase CF1 beta subunit	A0A0C5CEC7	atpB	SL	17	39	5.28/53467		7.5
***Oxidative stress***									
3	Superoxide dismutase [Cu-Zn]	K4AX22	SODC	SL	21	54	5.47/15285		0.6
10	Thioredoxin peroxidase 1	Q7Y240	TPx1	SL	11	64	5.18/17437		0.5
15	Glutathione S-transferase L3	K4D3M6	GSTL3	SL	7	26	5.06/27183		0.4
17	L-ascorbate peroxidase 2	K4CQB7	APX2	SL	16	46	5.63/27635		0.4
19	Glutathione-S-transferase φ class	K4C3T2	GSTF8	SL	29	61	5.98/23723		0.4
22	Ascorbate peroxidase	Q52QQ4	APX	SL	17	60	5.86/27322		0.6
23	Ascorbate peroxidase	Q52QQ4	APX	SL	4	14	5.86/27322		0.6
27	Lactoylglutathione lyase	K4B9T4	GLX1	SL	31	39	6.62/38542		0.6
***Stress and defense***									
2	Fruit-ripening protein	O82575	Asr1	SL	6	58	6.48/12555		0.4
4	17.6 KDa Class I sHSP	Q96489	HSP17.6	SP	32	44	6.32/17613		0.3
5	TSI-1 protein	O49881	TSI-1	SL	13	74	5.61/20221		0.5
6	Glycine-rich RNA-binding protein	K4B3H9	GRP1	SL	6	43	5.59/17343		0.3
7	17.8 KDa Class I sHSP	G5DGD2	HSP17.8	SL	40	56	5.82/17639		0.3
9	Chloroplast sHSP	Q95661	HSP21	SL	9	42	7.84/26227		0.5
11	Metacaspase 1	Q8H272	MCA1	SL	2	11	4.78/44864		0.4
13	Chloroplast sHsp	Q95661	HSP21	SL	7	29	7.84/26227		0.6
14	Chaperonin 21	K4DC13	CPN21	SL	23	53	5.32/22416		0.6
25	Embryo-abundant EMB	Q8S271	P0415C01.10	OS	4	31	6.60/33104		0.4
26	Protein phosphatase 2C	Q6QLU0	PP2C	SL	7	52	5.67/30944		7.2
33	Dehydrin ERD10	K4BVU7	ERD10	SL	6	37	5.12/23111		0.2
34	Heat shock protein 70 (fragment)	Q40151	MED37C	SL	7	13	5.17/71515		0.4
48	HSP70-interacting protein 1	K4CNT4	HIP1	SL	6	18	4.92/45598		0.2
49	Chaperonin-60 α-sub	K4DAD5	CPN60A1	SL	21	25	5.21/61955		0.2
50	Stress-induced protein STI-1-like protein	A0A1V1H194	OB_Ba0011H08.28	OB	32	61	6.49/57217		0.1
51	Heat shock protein 60	K4CWE4	CPN60	SL	30	45	5.52/61560		0.5
52	Chaperonin 60 beta subunit	K4AV63	CPN60B2	SL	9	18	5.72/62992		0.2
53	Heat shock protein 70	K4D9L9	MED37C	SL	23	30	5.10/71414		0.4
54	Heat shock protein 70	K4D9L9	MED37C	SL	33	40	5.10/71474		0.6
55	Chaperone protein ClpB3	K4BC16	CLPB3	SL	13	14	6.17/110377		0.2
56	Mitochondrial HSO70 2	K4B2I9	HSP70	SL	26	35	5.75/72970		0.1

Spot number, protein name, UniProtKB accession, gene name, organism, unique detected peptides, sequence coverage (%), theoretical pI and molecular mass values, and fold change (biochar vs. control) are shown. Proteins are reported according to their belonging to functional classes (stress and defense, oxidative stress, and energy/carbon metabolism). SL, Solanum lycopersicum; SP, Solanum peruvianum; OS, Oryza sativa; OB, Oryza brachyantha; AT, Arabidopsis thaliana; SC, Solanum chacoense; ST, Solanum tuberosum; SPE, Solanum pennellii; FA, Fragaria ananassa; DS, Datura stramonium.

### Carbon and Energy Metabolism

Most of the differentially represented proteins in the fruits of from tomato plants grown in soil amended with 5% biochar belong to the carbon/energy metabolism functional group, which included 17 unique protein entries (present in 26 spots). All resulted over-represented in the berries of biochar-treated plants. In tomato berries, glycolysis and respiration represent the main carbon and energetic fluxes that fuel substrates to sustain the respiratory burst as well as biosynthesis of cofactors, pigments, and metabolites during the maturation process (Livak and Schmittgen, 2001; Sarry et al., 2004). Interestingly, seven proteins involved in the oxidative phase of glycolysis were over-represented in the berries of biochar-treated plants, namely, cytosolic fructose-bisphosphate aldolase 6 (spot 39), triose phosphate isomerase cytosolic and chloroplastic isoforms (spots 16, 18 and 20), glyceraldehyde 3-phosphate dehydrogenase (spot 29), phosphoglycerate kinase (spot 38) and enolase (spots 37, 42, and 43). These findings suggest that biochar addition brought a stimulation of carbon catabolism during ripening, which resulted in improved growth and yield of mature berries. In agreement with above-mentioned observation, levels of cytosolic (cMDH) (spot 28 and 31) and NAD-dependent mitochondrial (mMDH) (spot 30) malate dehydrogenase were also increased in biochar-treated berries. MDH is a pivotal enzyme for regulation of malate concentration; its over-representation in biochar-challenged plants fairly correlates with their increased sugar metabolism during ripening as well as the stimulatory effect of this amendant on tomato growth and ripening. In fact, massive malate oxidation takes place during the last phase of maturation, in order to sustain the respiratory burst and to provide through the Krebs cycle carbon intermediates for secondary metabolites and volatiles biosynthesis, which accumulates in the mature berries (Carrari and Fernie, 2006). In line with augmented carbon metabolism in biochar-challenged plants were also the observed increased levels of UTP-glucose-1-phosphate uridylyltransferase (spot 41), and cytoplasmic (spot 21) and plastidial (spot 8) inorganic pyrophosphatase, which are involved in sucrose synthesis and thus promoted sucrose or hexose accumulation in treated plants (Carrari and Fernie, 2006).

In biochar-challenged plants, above-mentioned proteomic changes corresponded to a parallel over-representation of enzymes involved in photosynthetic carbon assimilation, namely, ribulose bisphosphate carboxylase large (spot 40) and small (spot 1) chain, and ribulose biphosphate carboxylase/oxygenase activase (spots 32, 35, and 36), and in (photosynthetic) energy production, e.g., ATP synthase β-subunit (spots 44 and 45), ATP synthase CF1 beta subunit (spots 46 and 47), and oxygen-evolving enhancer protein 1-2 (spot 24), which directly contributed in stabilizing/synthesizing molecules essential to provide the fruit energetic supply and to maintain the berry endogenous O_2_ balance. These data suggest that different energetic processes are activated in San Marzano as result of biochar addition to the soil, ultimately determining improved growth and yield of mature berries.

### Stress and Defense

Additional differentially represented proteins categorized in the broad functional group of stress- and defense-related proteins, for a whole of 27 unique protein entries (present in 30 spots). In particular, seven components of the cellular antioxidant machinery showed down-represented levels in biochar-treated plants, namely, (Cu-Zn) superoxide dismutase (spot 3), two isoforms of ascorbate peroxidase (spot 17, 22, and 23), glutathione-S-transferase L3 and φ class (spot 15 and 19), thioredoxin peroxidase 1 (spot 10), and lactoylglutathione lyase (spot 27). In mature berries, antioxidant enzymes are abundant proteins since fruit ripening is generally paralleled by an increase of oxidative metabolism, with accumulation of H_2_O_2_ and reactive oxigen species (ROS) as well as of membrane lipid peroxidation (Carrari and Fernie, 2006). This ROS accumulation is often balanced by the activity of different cellular antioxidant enzymes and scavenging systems, such as catalases, superoxide dismutases, and the ascorbate-gluthathione cycle. In this context, superoxide dismutases and ascorbate peroxidases are the major enzymes deputed to H_2_O_2_ removal from the plant cell (Jimenez et al., 2002). Besides detoxification of xenobiotics, plant glutathione-S-transferases also function as GSH-dependent peroxidases, which reduce organic hydroproxides to monohydroxyalcohols, thus limiting oxidative injury (Rocco et al., 2006). On the other hand, thioredoxin peroxidase is a key component for the control of mitochondrial H_2_O_2_ metabolism (Huang et al., 2007), while lactoylglutathione lyase participates into the glutathione-based detoxification of methylglyoxal that is produced by carbohydrate and lipid metabolism (Jimenez et al., 2002). On the overall, our result demonstrate that biochar treatment of San Marzano plants experiencing low-input farming conditions determined a significant reduction of antioxidant enzymes, which may suggest the occurrence of other compensative mechanisms to face the augmented levels of ROS generally observed during fruit ripening.

A number of other components involved in the plant response to stress, which belong to the heat shock protein (HSP) or chaperonin protein families, were also detected as down-represented in tomato plants challenged with biochar. They were 17.6 (spot 4) and 17.8 (spot 7) kDa class I sHSPs, chloroplast sHSP (spot 9 and 13), two HSP70 isoforms (spot 34, 53, and 54), mitochondrial HSO70 2 (spot 56), HSP-interacting protein 1 (spot 48), HSP 60 (spot 51), chaperonin 21 (spot 14), chaperone protein ClpB3 (spot 55), and chaperonin 60 (spot 49) and β (spot 52) subunits. While functional characterization of chaperonins in plants is quite limited, more information is available on HSPs. The latter are a family of ubiquitous molecular chaperones whose main function is to prevent protein aggregation during stress, which group into five classes according to their molecular mass (Jacob et al., 2017). sHSPs are the most prevalent in plants, where they are expressed in distinct cellular compartments not only in response to a wide range of environmental stresses, including the oxidative one, but also during fruit development and maturation (Paull and Jung Chen, 2000). Biochar treatment of tomato plants brought about a consistent reduction in the abundance of HPSs and chaperonins in the corresponding berries, ranging from about 50% to 80%, as compared to untreated counterparts.

Finally, other proteins involved in the plant response to different stress conditions were also ascertained with decreased concentration levels in tomato plants treated with biochar, namely, fruit-ripening protein (spot 2), TSI-1 protein (spot 5), embrio-abundant EMB (spot 25), dehydrin ERD10 (spot 33), stress-induced protein sti1-like protein (spot 50), glycine-rich RNA-binding protein (spot 6), and metacaspase 1 (spot 11). Fruit-ripening protein, embrio-abundant EMB, dehydrin ERD10, and stress-induced protein STI-1-like protein are molecules generally related to plant response to various environmental stresses. In particular, fruit-ripening protein shares 90% sequence identity with abscisic acid stress ripening protein 1, a nuclear protein that interacts with chromatin and accumulates in leaves in response to water stress and in fruits during maturation (Rocco et al., 2006). Analogously, glycine-rich RNA-binding protein, TSI-1 protein and metacaspase 1 are generally involved in plant response to biotic stresses. In particular, metacaspase 1 belongs to the type I class of putative cysteine proteases, which are distantly related to caspases (Rocco et al., 2006). In plants, metacaspases have been shown to act as cell death regulators in the hypersensitive response (HR) to pathogen attack (Lema Asqui et al., 2018).

In the whole, above-reported data demonstrate that the addition of 5% biochar to San Marzano low-input cultivation brought a general and consistent down-representation of proteins involved in the response of plants to abiotic and (to a reduced extent) biotic stresses. Even though conflicting data exist as far as the biochar-mediated modulation of stress and defense response in plants, our proteomic results are in line with gene expression studies on *Arabidopsis* and lettuce treated with biochar (Viger et al., 2015), where a general up-regulation of growth-promoting genes and a down-regulation of stress and defense ones occurred following administration of this soil amendant.

### RT-qPCR Analysis of Jasmonic Acid-Related Genes

**Figure 5:**
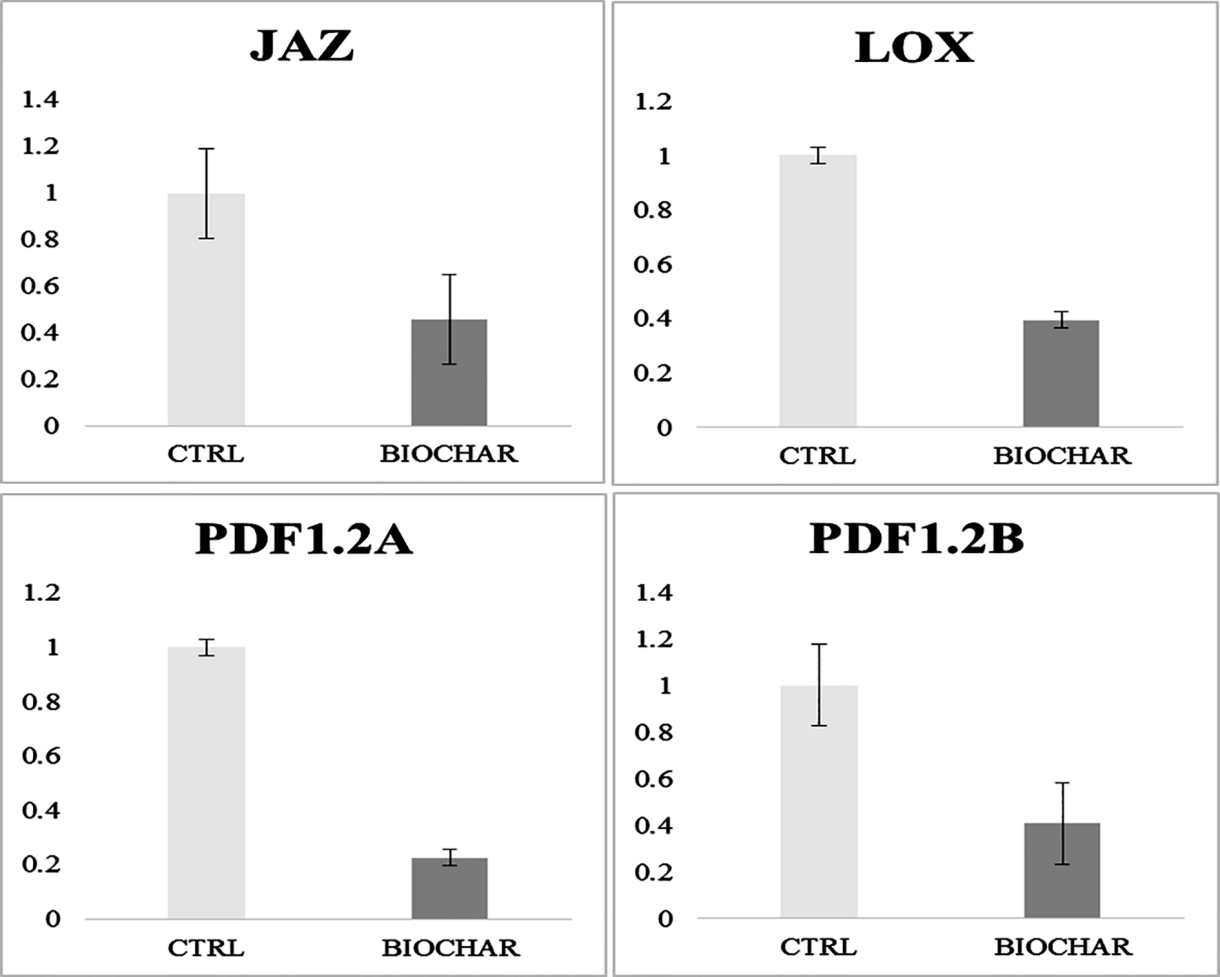
Gene expression analysis in fruits from tomato plants experiencing low-input farming conditions in soils amended with or not with 5% Verfofood biochar. Reported are data on genes involved in JA pathway, namely, plant defensin factor 1.2A and 1.2B (PDF1.2A and PDF1.2B, respectively), lipoxygenase (LOX) and jasmonate-zim domain (JAZ). Data were analyzed with the 2-ΔΔCt method.

Recently, microarray investigations have suggested that genes related to the signaling pathway of the defense response hormone jasmonic acid (JA) are down-regulated in biochar-treated plants (Viger et al., 2015). Since we did not recorded here by proteomics any evidence regarding the modulation of proteins related to above-mentioned genes, we extended our analysis to verify the accordance of the model of San Marzano plants experiencing low-input farming conditions together with administration of 5% biochar with that of the growth-defense trade-off model mentioned above (Viger et al., 2015). To this purpose, a RT-qPCR analysis of genes related to JA-modulated pathways was carried out. Results shown in **Figure 5** demonstrate that biochar treatment in our case determined a down-regulation of plant defensin PDF1.2A, defensin PDF1.2B, and lipoxygenase (LOX) genes in tomato berries, whereas a repressor gene of the JA response pathway, namely, iasmonate-zim domain protein (JAZ), was up-regulated. These results are in agreement with that already reported by Viger and coworkers for *Arabidopsis* and lettuce plants.

## Conclusion

Nowadays, agronomic data on the effects of biochar administration on tomato growth, yield, and fruit quality are scarce and contrasting (Vaccari et al., 2015; Polzella et al., 2019), whereas those on related molecular information are practically absent. The effectiveness of biochar treatments very likely depend on the characteristics of the soil to which it is added, as well as on the farming conditions (use or not of fertilizers) generally adopted. Best effects are usually obtained with acid and low nutrients soils; in fact, biochar administration generally increases soil pH value and modifies soil characteristics to improve water retention and nutrient availability (N, P, and K). In field agronomic studies on tomato cultivars, Vaccari and coworkers reported that biochar addition to a fertile soil improves tomato growth but not yield (Vaccari et al., 2015), whereas Polzella and colleagues showed that biochar administration to plants growing in a neutral and low-input (for nutrients) soil did not significantly ameliorate growth and yield performances (Polzella et al., 2019). Under our farming conditions (slightly acidic pH value of the soil and no use of fertilizers), addition of 5% biochar to San Marzano tomato plants modified soil characteristics, at the same time significantly affecting vegetative growth and fruit yield. This fact confirms that the outcome of the treatment with this amendant is more dependent on agronomic site features than on crop species and/or biochar type (Jay et al., 2015). At the molecular level, unique reference studies are the above-mentioned ones on *Arabidopsis* and lettuce, which were performed by a gene expression platform on plants grown in a soil treated or not with biochar (Viger et al., 2015). These investigations identified auxin and brassinosteroid signaling pathways (Camoni et al., 2018) as key determinants for the growth-promoting effect of biochar. They also proposed a growth-defense trade-off model, where up-regulation of growth-promoting genes is accompanied by down-regulation of a large set of plant defense genes. The proteomic and gene expression results presented in this study are in line with this model, since plant treatment with biochar determined an opposite quantitative trend of genes related to the last phase of reproductive growth (ripening) and of genes related to stress and defense responses. In conclusion, our data suggest that biochar administration under controlled farming conditions can be a useful sustainable practice to improve tomato growth and yield. At molecular level, besides amelioration of water and nutrient availability, this study suggests that the biochar growth-promoting effect is underwent by the activation of specific signaling pathways and molecular mechanisms that deserve a dedicated deeper analysis.

## Data Availability Statement

The raw data supporting the conclusions of this article will be made available by the authors, without undue reservation, to any qualified researcher.

## Author Contributions

MT and MR were responsible for design of and planning the experiments, analyzed data, and drafted manuscript. MT and SA performed experiments. MM, AS, and MR interpreted results of experiments, edited, and revised manuscript. MR approved final version of manuscript.

## Conflict of Interest

The authors declare that the research was conducted in the absence of any commercial or financial relationships that could be construed as a potential conflict of interest.

## References

[B1] Akhter A. Hage-Ahmed K. Soja G. Steinkellner S. (2015). Compost and biochar alter mycorrhization, tomato root exudation, and development of Fusarium oxysporum f. sp. lycopersici. Front. Plant Sci. 6, 529 1–52913. 10.3389/fpls.2015.00529 26217373 PMC4498038

[B2] Auffhammer M. Ramanathan V. Vincent J. R. (2012). The monsoon, and rice yield in India. Climate Change 111, 411–424. 10.1007/s10584-011-0208-4

[B3] Biederman L. A. Stanley Harpole W. (2013). Biochar and its effects on plant productivity and nutrient cycling: A meta-analysis. GCB Bioenergy 5, 202–214. 10.1111/gcbb.12037

[B4] Camoni L. Visconti S. Aducci P. Marra M. (2018). 14-3-3 proteins in plant hormone signaling: doing several things at once. Front. Plant Sci. 9, 297. 10.3389/fpls.2018.00297 29593761 PMC5859350

[B5] Carrari F. Fernie A. R. (2006). Metabolic regulation underlying tomato fruit development. J. Exp. Bot. 57, 1883–1897. 10.1093/jxb/erj020 16449380

[B6] Copley T. Bayen S. Jabaji S. (2017). Biochar Amendment Modifies Expression of Soybean and Rhizoctonia solani Genes Leading to Increased Severity of Rhizoctonia Foliar Blight. Front. Plant Sci. 8, 221. 10.3389/fpls.2017.00221 28270822 PMC5318381

[B7] Elad Y. Cytryn E. Meller Harel Y. Lew B. Graber E. R. (2011). The biochar effect: Plant resistance to biotic stresses. Phytopathol. Mediterr. 50, 335–349.

[B8] Elmer W. H. Pignatello J. J. (2011). Effect of biochar amendments on mycorrhizal associations and fusarium crown and root rot of asparagus in replant soils. Plant Dis. 95, 960–966. 10.1094/PDIS-10-10-0741 30732119

[B9] Ercolano M. R. Carli P. Soria A. Cascone A. Fogliano V. Frusciante L. (2008). Biochemical, sensorial and genomic profiling of traditional Italian tomato varieties. Euphytica 164, 571–582. 10.1007/s10681-008-9768-4

[B10] Ercolano M. R. Sacco A. Ferriello F. D’Alessandro R. Tononi P. Traini A. (2014). Patchwork sequencing of tomato San Marzano and Vesuviano varieties highlights genome-wide variations. BMC Genomics 15, 138. 10.1186/1471-2164-15-138 24548308 PMC3936818

[B11] Harel Y. M. Elad Y. Rav-David D. Borenstein M. Shulchani R. Lew B. (2012). Biochar mediates systemic response of strawberry to foliar fungal pathogens. Plant Soil 350, 211–242. 10.1007/s11104-012-1129-3

[B12] Huang R. Xia R. Hu L. Lu Y. Wang M. (2007). Antioxidant activity and oxygen-scavenging system in orange pulp during fruit ripening and maturation. Sci. Hortic. 113, 166–172. 10.1016/j.scienta.2007.03.010

[B13] Huang Q. Wang Y. Li B. Chang J. Chen M. Li K. (2015). TaNAC29, a NAC transcription factor from wheat, enhances salt and drought tolerance in transgenic Arabidopsis. BMC Plant Biol. 15, 268–282. 10.1186/s12870-015-0644-9 26536863 PMC4632686

[B14] Jacob P. Hirt H. Bendahmane A. (2017). The heat-shock protein/chaperone network and multiple stress resistance. Plant Biotechnol. J. 15, 405–414. 10.1111/pbi.12659 27860233 PMC5362687

[B15] Jay C. N. Fitzgerald J. D. Hipps N. A. Atkinson C. J. (2015). Why short-term biochar application has no yield benefits: evidence from three field-grown crops. Soil Use Manage. 31, 241–250. 10.1111/sum.12181

[B16] Jeffery S. Verheijen F. G. A. van der Velde M. Bastos A. C. (2011). A quantitative review of the effects of biochar application to soils on crop productivity using meta-analysis. Agric. Ecosyst. Environ. 144, 175–187. 10.1016/j.agee.2011.08.015

[B17] Jimenez A. Creissen G. Kular B. Firmin J. Robinson S. Verhoeyen M. (2002). Changes in oxidative processes and components of the antioxidant system during tomato fruit ripening. Planta 214, 751–758. 10.1007/s004250100667 11882944

[B18] Keech O. Carcaillet C. Nilsson M. C. (2005). Adsorption of allelopathic compounds by wood-derived charcoal: the role of wood porosity. Plant Soil 272, 291–300. 10.1007/s11104-004-5485-5

[B19] Lafferty K. D. (2009). The ecology of climate change and infectious diseases. Ecology 90, 888–900. 10.1890/08-0079.1 19449681

[B20] Laghari M. Naidu R. Xiao B. Hu Z. Mirjat M. S. Hu M. (2016). Recent developments in biochar as an effective tool for agricultural soil management: a review. J. Sci. Food Agric. 96, 4840–4849. 10.1002/jsfa.7753 27116042

[B21] Laird D. A. Brown R. C. Amonette J. E. Lehmann J. (2009). Review of the pyrolysis platform for coproducing bio-oil and biochar. Biofuel Bioprod. Bior. 3, 547–562.[7]. 10.1002/bbb.169

[B22] Laird D. A. Fleming P. Davis D. D. Horton R. Wang B. Karlen D. L. (2010). Impact of biochar amendments on the quality of a typical Midwestern agricultural soil. Geoderma 158, 443–449. 10.1016/j.geoderma.2010.05.013

[B23] Lehmann J. (2007a). A handful of carbon. Nature 447, 143–144. 10.1038/447143a 17495905

[B24] Lehmann J. (2007b). Bio-energy in the black. Front. Ecol. Environ. 5, 381–387. 10.1890/1540-9295(2007)5[381:BITB]2.0.CO;2

[B25] Lema Asqui S. Vercammen D. Serrano I. Valls M. Rivas S. Van Breusegem F. (2018). AtSERPIN1 is an inhibitor of the metacaspase AtMC1-mediated cell death and autocatalytic processing in planta. New Phytol. 218, 1156–1166. 10.1111/nph.14446 28157265

[B26] Livak K. J. Schmittgen T. D. (2001). Analysis of relative gene expression data using real-time quantitative PCR and the 2^–ΔΔ^*^C^* method. Methods 25, 402–408. 10.1006/meth.2001.1262 11846609

[B27] Marris E. (2006). Putting the carbon back: black is the new green. Nature 442, 624–626. 10.1038/442624a 16900176

[B28] Mehari Z. H. Elad Y. Rav-David D. Graber E. R. Meller Harel Y. (2015). Induced systemic resistance in tomato (Solanum lycopersicum) against Botrytis cinerea by biochar amendment involves jasmonic acid signaling. Plant Soil 395, 31–44. 10.1007/s11104-015-2445-1

[B29] Méndez A. Terradillos M. Gascó G. (2013). Physicochemical and agronomic properties of biochar from sewage sludge pyrolysed at different temperatures. J. Anal. Appl. Pyrolysis 102, 124–130. 10.1016/j.jaap.2013.03.006

[B30] Negri V. (2003). Landraces in central Italy: Where and why they are conserved and perspectives for their on-farm conservation. Genet. Resour. Crop Evol. 50, 871–885. 10.1023/A:1025933613279

[B31] Novak J. M. Busscher W. J. Laird D. L. Ahmedna M. Watts D. W. Niandou M. A. S. (2009). Impact of biochar amendment on fertility of a southeastern coastal plain soil. Soil Sci. 174, 105–112. 10.1097/SS.0b013e3181981d9a

[B32] Paiardini A. Aducci P. Cervoni L. Cutruzzolà F. Di Lucente C. Janson G. (2014). The phytotoxin fusicoccin differently regulates 14-3-3 proteins association to mode III targets. IUBMB Life 66, 52–62. 10.1002/iub.1239 24408864

[B33] Park J. H. Choppala G. K. Bolan N. S. Chung J. W. Chuasavathi T. (2011). Biochar reduces the bioavailability and phytotoxicity of heavy metals. Plant Soil 348, 439–451. 10.1007/s11104-011-0948-y

[B34] Paull R. E. Jung Chen N. (2000). Heat treatment and fruit ripening. Postharvest Biol. Technol. 21, 21–37. 10.1016/S0925-5214(00)00162-9 11543413

[B35] Polzella A. De Zio E. Arena S. Scippa G. S. Scaloni A. Montagnoli A. (2019). Toward an understanding of mechanisms regulating plant response to biochar application. Plant Biosyst. 159, 163–172. 10.1080/11263504.2018.1527794

[B36] Rocco M. D’Ambrosio C. Arena S. Faurobert M. Scaloni A. Marra M. (2006). Proteomic analysis of tomato fruits from two ecotypes during ripening. Proteomics 6, 3781–3791. 10.1002/pmic.200600128 16739135

[B37] Sant’Ana D. V. P. Lefsrud M. (2018). Tomato proteomics: tomato as a model for crop proteomics. Sci. Hortic. 239, 224–233. 10.1016/j.scienta.2018.05.041

[B38] Sarry J. E. Sommerer N. Sauvage F. X. Bergoin A. Rossignol M. Albignac G. (2004). Grape barry biochemesty revisited upon proteomic analysis of the mesocarp. Proteomic 4, 201–215. 10.1002/pmic.200300499 14730682

[B39] Shoaf N. Hoagland L. Egel D. (2016). Suppression of phytophthora blight in sweet pepper depends on biochar amendment and soil type. Hort. Sci. 51, 518–524. 10.21273/HORTSCI.51.5.518

[B40] Spokas K. A. Reicosky D. C. (2009). Impacts of sixteen different biochars on soil greenhouse gas production. Ann. Environ. Sci. 3, 179–193.

[B41] Spokas K. A. Novak J. M. Stewart C. E. Cantrell K. B. Uchimiya M. DuSaire M. G. (2011). Qualitative analysis of volatile organic compounds on biochar. Chemosphere 85, 869–882. 10.1016/j.chemosphere.2011.06.108 21788060

[B42] Steinbeiss S. Gleixner G. Antonietti M. (2009). Effect of biochar amendment on soil carbon balance and soil microbial activity. Soil Biol. Biochem. 41, 1301–1310. 10.1016/j.soilbio.2009.03.016

[B43] Vaccari F. P. Maienza A. Miglietta F. Baronti S. Di Lonardo S. Giagnoni L. (2015). Biochar stimulates plant growth but not fruit yield of processing tomato in a fertile soil. Agric Ecosyst. Environ. 207, 163–170. 10.1016/j.agee.2015.04.015

[B44] Van Zwieten L. Kimber S. Morris S. Downie A. Berger E. Rust J. (2010a). Influence of biochars on flux of N2O and CO2from Ferrosol. Aust. J. Soil Res. 48, 555–568. 10.1071/SR10004

[B45] Van Zwieten L. Kimber S. Downie A. Morris S. Petty S. Rust J. (2010b). A glasshouse study on the interaction of low mineral ash biochar with nitrogen in a sandy soil. Aust. J. Soil Res. 48, 569–576. 10.1071/SR10003

[B46] Viger M. Hancock R. D. Miglietta F. Taylor G. (2015). More plant growth but less plant defence? First global gene expression data for plants grown in soil amended with biochar. GCB Bioenergy 7, 658–672. 10.1111/gcbb.12182

